# Emission characteristics of harmful air pollutants from cremators in Beijing, China

**DOI:** 10.1371/journal.pone.0194226

**Published:** 2018-05-02

**Authors:** Yifeng Xue, Linglong Cheng, Xi Chen, Xiaoman Zhai, Wei Wang, Wenjie Zhang, Yan Bai, Hezhong Tian, Lei Nie, Shihao Zhang, Tong Wei

**Affiliations:** 1 National Engineering Research Center of Urban Environmental Pollution Control, Beijing Municipal Research Institute of Environmental Protection, Beijing, China; 2 Key Laboratory of Pollution Control of Ministry of Civil Affairs, 101 Institute of Ministry of Civil Affairs, Beijing, China; 3 Chinese Research Academy of Environmental Sciences, Beijing, China; 4 Babaoshan funeral parlor, Beijing, China; 5 State Key Joint Laboratory of Environmental Simulation & Pollution Control, School of Environment, Beijing Normal University, Beijing, China; 6 College of Resource Environment and Tourism, Capital Normal University, Beijing, China; National Sun Yat-sen University, TAIWAN

## Abstract

The process of corpse cremation generates numerous harmful air pollutants, including particulate matter (PM), sulfur dioxide (SO_2_), nitrogen oxides (NO_x_), volatile organic compounds (VOCs), and heavy metals. These pollutants could have severe effects on the surrounding environment and human health. Currently, the awareness of the emission levels of harmful air pollutants from cremators and their emission characteristics is insufficient. In this study, we obtained the emission characteristics of flue gas from cremators in Beijing and determined the localized emission factors and emission levels of harmful air pollutants based on actual monitoring data from nine typical cremators. The results show that the emissions of air pollutants from the cremators that directly discharge flue gas exceed the emission standards of China and Beijing. The installation of a flue gas post-treatment system could effectively reduce gaseous pollutants and the emission levels of PM. After being equipped with a flue gas post-treatment system, the emission concentrations of PM_10_, PM_2.5_, CO, SO_2_ and VOCs from the cremators are reduced by 97.6, 99.2, 19.6, 85.2 and 70.7%, respectively. Moreover, the emission factors of TSP, PM_10_, PM_2.5_, CO, SO_2_ and VOCs are also reduced to 12.5, 9.3, 3.0, 164.1, 8.8 and 19.8 g/body. Although the emission concentration of VOCs from the cremators is not high, they are one of major sources of “odor” in the crematories and demand more attention. Benzene, a chemical that can seriously harm human health, constitutes the largest proportion (~50%) of the chemical components of VOCs in the flue gas from the cremators.

## Introduction

China has the highest annual number of deaths in the world. According to the “China Civil Affairs Statistical Yearbook 2015”, China’s national death toll was 9.77 million, and its corpse cremation rate was 47% in 2014. The process of corpse cremation generates numerous harmful air pollutants [1–3], including particulate matter (PM), SO_2_, NO_x_, CO, HCl, HF, NH_3_, VOCs, heavy metals, polychlorinated dibenzo-p-dioxins and dibenzofurans (PCDD/Fs) [4–8]. Due to the characteristics of the funeral sector, the chimney heights are usually low, and the air pollutants disperse close to the ground, thus severely affecting the surrounding air quality and human health [9–17]. The problem of the emissions of harmful air pollutants from cremators is causing increasing social concern.

To strengthen the control and management of pollutant emissions from cremators and incinerators, China and Beijing have issued emission standards of air pollutants for crematories (GB13801-2015 and DB1203-2015). These standards have enhanced the emission limits of air pollutants from cremators and incinerators and clarified relevant requirements on pollution control, which are promoting the implementation of prevention and control measures in the crematories to reduce pollutant emission levels. However, the standards have not specified the emission limits of PM_10_, PM_2.5_ and VOCs from cremators. Previous studies on air pollutants from cremators have typically focused on the problem of emissions of PCDD/Fs and other persistent pollutants in China and other countries [5,18–20]. The emission characteristics of PM_10_, PM_2.5_ and VOCs in flue gas from cremators have seldom been reported. These pollutants have provoked increasing attention for their severe impacts on air quality, visibility and human health. The EU EMEP/EEA guidebook (2016) [21] provided the emission factors of pollutants such as PM_10_, PM_2.5_ and VOCs from cremators, but this guidance did not distinguish the cremators with and without flue gas purification systems, and the collected data were not timely enough to accurately represent the current emission levels from cremators. In China, relevant studies have primarily focused on quantifying conventional pollutants such as total suspended particulates (TSP), SO_2_, NO_x_, CO and persistent organic pollutants from cremators [10,11,22]. Research into the emission concentrations and emission factors of fine particulate matter and VOCs from cremators is relatively scant.

To better understand the emissions of flue gas from cremators after the implementation of the standards in China, we examined the emission levels and emission characteristics of PM (TSP, PM_10_ and PM_2.5_) and air pollutants (SO_2_, NO_x_, CO and VOCs) from different types of cremators with and without flue gas post-treatment systems by practical monitoring of nine crematories in Beijing. We determined the localized emission factors and analyzed the chemical components of VOCs in the flue gas from these crematories. This study was the first in China to monitor and analyze PM_10_, PM_2.5_, VOCs and their chemical components as well as quantify the pollutant emission levels from the cremators. The result could provide a reference for the subsequent assessment and revision of national or local standards and serve as a reference providing support for the current civil administration and environmental management.

## Materials and methods

### Study objects

Beijing, the capital of China, is located in the northern part of the North China Plain, covering an area of 16,410.54 km^2^. It is characterized by high residential density, with a resident population of 21.516 million. Limited in land resources, cremation is implemented as a fundamental national policy. Beijing has achieved a cremation rate of nearly 100% for many years. There are currently 12 funeral parlors in Beijing; two of them are located in the urban area, i.e., Babaoshan funeral parlor and Dongjiao funeral parlor, and the others are located in the suburbs. Each funeral parlor is for corpse cremation.

Based on the site survey and data collection at 12 funeral parlors in Beijing, we present the following findings. Regarding fuel type, the cremators in Beijing were mainly oil-fired, and the fuel conversion from oil to gas was performed for only 15 cremators in the Babaoshan funeral parlor. In terms of furnace structure, Beijing’s cremators were predominantly car-bottom type with a percentage of ~70%. Considering end-of-pipe control, 59.8% of the cremators in Beijing were equipped with a flue gas purification system, which mainly included flue gas cooling, deacidification, deodorization and dedusting devices. The flue gas containing various harmful air pollutants emitted from combustion has a high temperature. To prevent the re-synthesis of dioxins, the flue gas is rapidly cooled to avoid recombination. Moreover, to remove the acid gases such as SO_2_ and H_2_S in the flue gas, alkali liquor is used to neutralize the acid gas, and the activated carbon is used to adsorb VOCs and odor components. Finally, the particulate matter in the flue gas is removed by a dust collector, thereby reducing the concentration of harmful air pollutants in the flue gas. Based on the control measure equipment for pollutants emitted from the cremators, the furnace type and fuel type, one typical cremator was selected from each of nine selected funeral parlors (geographical coordinates information in S1 Table) for monitoring the actual flue gas emissions. The emission concentrations of air pollutants were sampled, and the emission factors were determined. The configuration of the nine cremators is depicted in **Table 1**. Four cremators post-processed the flue gas, and five discharged the flue gas directly. There was one gas-fired cremator and eight oil-fired cremators.

**Table 1:** Configuration of nine cremators.

Facility No.	A	B	C	D	E	F	G	H	I
Furnace type	Car-bottom	Car-bottom	Car-bottom	Flat plate	Flat plate	Car-bottom	Car-bottom	Car-bottom	Car-bottom
Dust collector	Bag filter	Bag filter	Bag filter	Bag filter	×	×	×	×	×
Secondary chamber	○	○	○	○	×	○	○	○	○
Flue gas cooling device	Air cooling	Air cooling	Air cooling	Air cooling	×	×	×	×	×
Deacidification device	○	○	○	○	×	×	×	×	×
Fuel	Natural gas	oil	oil	oil	oil	oil	oil	oil	oil

**Note:** ○ contains the device, × does not contain the device.

### Sample collection

**Fig 1:**
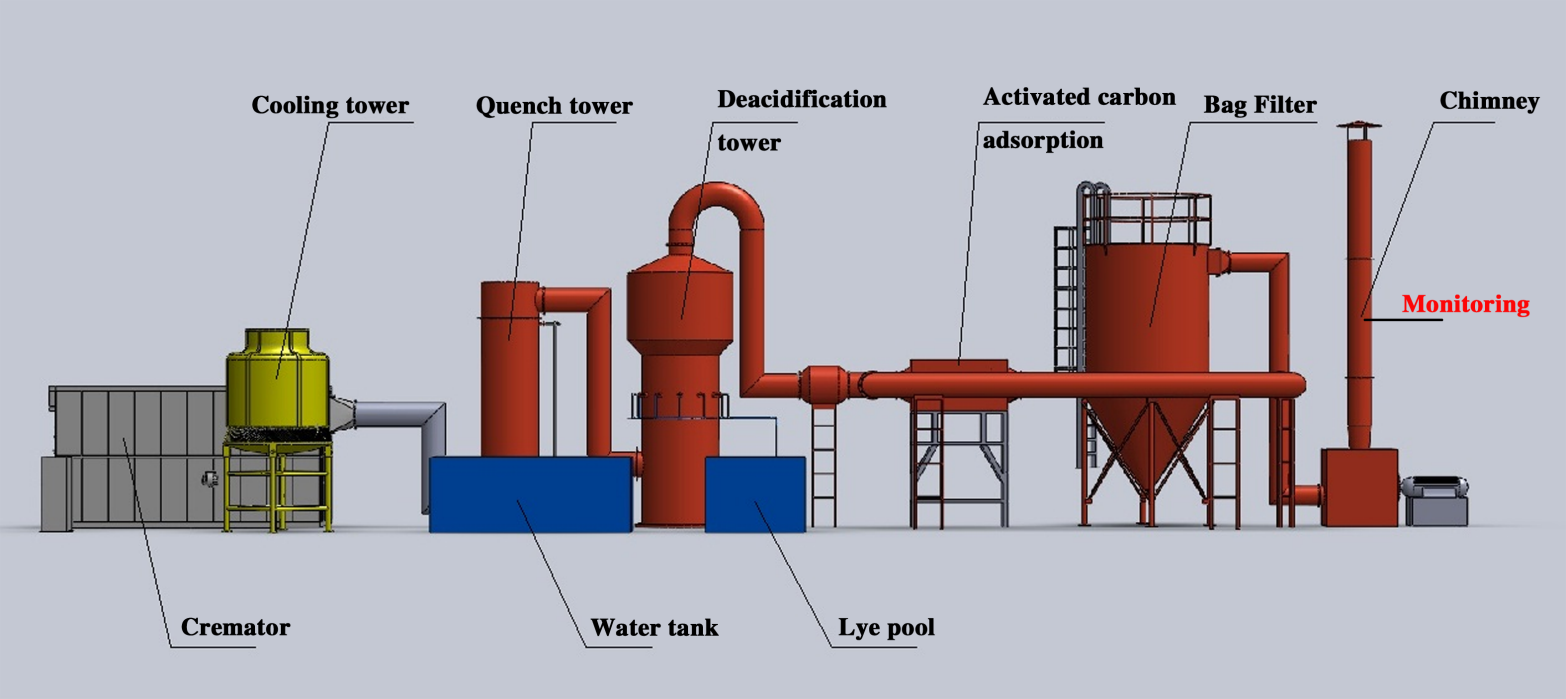
Typical flue gas post-treatment system of a cremator.

The sampling and monitoring of PM and air pollutants were conducted from the nine selected cremators during the winter from November 2016 to January 2017. The sampling location was on the exhaust stack, which can be seen in **Fig 1**. Dust samples (TSP, PM_10_ and PM_2.5_) were collected using a two-stage PM_10_ and PM_2.5_ virtual impactor (Model IV501, China) according to the method of ISO 13271:2012. Before sampling, we measured the temperature, water content, oxygen content, pressure, flow rate and other parameters at the sampling point to calculate the flow rate and the PM_2.5_ main flow, PM_2.5_ secondary flow and PM_10_ secondary flow required for estimating the sample nozzle diameter. The sampling period started from the beginning of corpse incineration in the main combustion chamber and ended when ashes were emptied from the main combustion chamber after the completion of the cremation process. Each cremator was sampled three times; unfortunately, due to pump failure during the sampling, 22 groups of available PM samples were obtained. Before sampling, a Teflon membrane was placed in a constant temperature and humidity chamber for 24 h of equilibration and then weighed with a precision electronic balance (0.00001 g resolution). After sampling, the Teflon membrane was held at constant temperature and humidity for 24 h and then weighed and stored. VOCs were sampled in air bags (10 L) from the flue gas of the cremators via a vacuum box and suction pump (set at 0.2 L/min) following the standard for emissions from stationary sources of volatile organic compounds using the Bags method (HJ 732–2014); the sampling time covered the time to cremate an entire body, which was approximately 45 minutes. Every cremator was sampled three times. In total, 27 groups of VOC samples were obtained from the cremators. The samples were stored in the dark and analyzed as soon as possible. Simultaneously, a microcomputer dust parallel sampler (TH880F, Tianhong, Wuhan) was applied to monitor the concentrations of CO, SO_2_ and NO_x_ and record the information of other parameters including flue gas temperature, humidity, oxygen content and flow rate. The trace gases (e.g., CO, SO_2_ and NO_X_) were determined by the fixed potential electrolysis method (HJ 693–2014 and HJ/T 57–2010).

### Concentration and chemical composition analysis of VOCs

The VOC samples were subjected to non-methane hydrocarbon (NMHC) analysis using a gas chromatograph (Beifen SP-3420A, China). The composition of the collected VOC samples was analyzed by a pre-concentrator (Entech 7100A, USA) and a gas chromatograph-mass spectrometer (Agilent 7890A-5975C, USA). The GC-MS enables qualitative and quantitative analysis of more than 100 types of VOCs according to the standard of stationary source emission determination of VOCs based on the sorbent adsorption and thermal desorption gas chromatography-mass spectrometry method (HJ 734–2014).

VOCs were identified based on their retention times and mass spectra and quantified by external calibration. The calibration standards were prepared by dynamically diluting the 100 ppbv Photochemical Assessment Monitoring Station (PAMS) standard mixture (57 NMHCs) and TO-15 standard mixture (65 compounds, from Spectra Gases Inc., NJ, USA) to 2.5, 5, 10, and 20 ppbv, with pure nitrogen as the mixing medium in a chamber after passing mass flow controllers. The calibration curves were obtained by running the four diluted standards plus humidified zero air in the same way as the field samples [23].

### Quality assurance and quality control (QA/QC)

The collected filter samples of particulate matter were collected on aluminum foil paper as soon as possible after sampling and stored in a refrigerator before analysis. The storage temperature was approximately -18°C. Weighing, extraction and analysis of the sample filter were performed in a closed and clean laboratory, which avoids errors introduced by the dust falling into the sample film during the experiment.

As for VOCs, before sampling, all canisters were cleaned at least five times by repeatedly filling and evacuating with humidified zero air. To check if there was any contamination in the canisters, after the cleaning procedure, all vacuumed canisters were re-filled with humidified zero air and stored in the laboratory for at least 24 h. They were analyzed using the same method as the field samples to ensure that none of the target VOC compounds were present in detectable amounts.

The precision of the VOCs measurements was compound-specific and within 3% for NMHCs and 6% for VOCs. The measurement accuracy was determined by treating the system with the dynamically diluted authentic standards and calculating the differences between the measured and true values. When running the samples, the system was challenged with a standard each day. If the reported values were beyond +/−10% of the standard values, recalibration of the system was performed [23].

### Calculation of emission factors

The emission factors of harmful air pollutants from the cremators were calculated based on their emission concentrations, flue gas amount and cremation time. The formulae are as follows:

$$E=\frac{C\times T\times V\times S\times 60}{1000}$$ (1)

$$S=\frac{\pi D^{2}}{4}$$ (2)

where *E* is the pollutant emission factor, g/body; *C* is the pollutant emission concentration, mg/m^3^; *T* is the cremation time, min; *V* is the flue gas flow speed, m/s; *S* is the cross-sectional area of the flue, m^2^; and *D* is the stack diameter, m.

## Results and discussion

### Emission concentrations of flue gas from cremators

**Fig 2:**
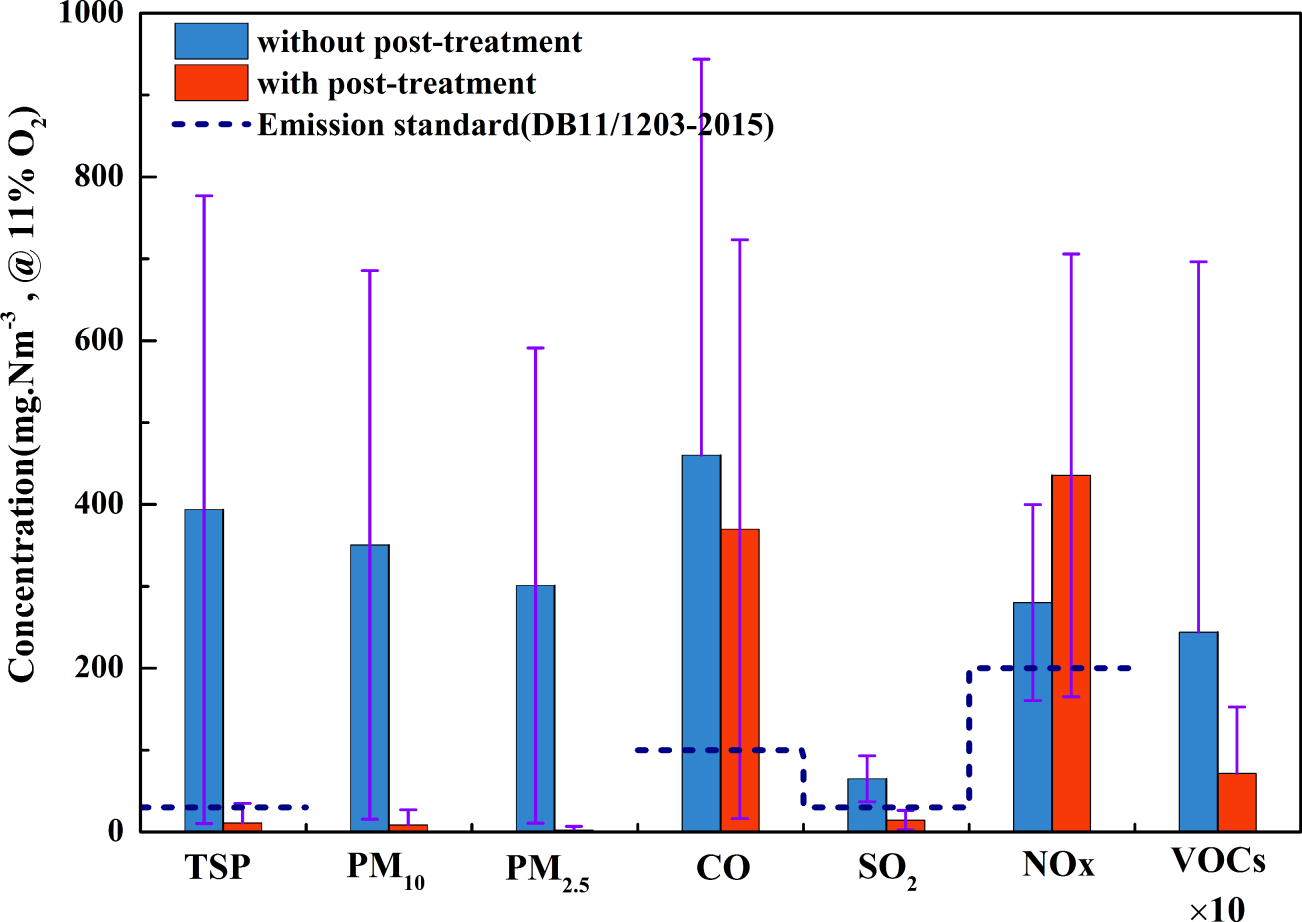
Emission concentration of harmful air pollutants from cremators.

The emission concentrations of harmful air pollutants from cremators are affected by various factors, such as fuel type, cremator type, flue gas post-treatment system and operational maintenance. In this study, monitoring was conducted for the concentrations of PM (TSP, PM_10_ and PM_2.5_) and gaseous pollutants (SO_2_, NO_x_, CO and VOCs) emitted from nine typical cremators (four with flue gas post-treatment devices) in Beijing as well as the related parameters (flue gas oxygen content, temperature, humidity and flow rate). The results (data in S2 Table) are presented in **Fig 2**.

PM from cremators is predominantly generated as a result of the incomplete combustion of the fuel or corpse. At the initial stage of combustion, the furnace temperature is relatively low and there is no guarantee for the retention time of the flue gas in the secondary combustion chamber, thus resulting in a relatively high concentration of dust discharged directly from the cremator without a dust removal treatment system. The emission concentration of TSP from those cremators without flue gas post-treatment systems ranged from 104.8 to 1,323.5 mg Nm^-3^ (@ 11% O_2_; the same as below), with an average concentration of 393.7 mg Nm^-3^, which greatly exceeded the emission limit for PM specified by local standards (30 mg Nm^-3^). Remarkably, the emission concentrations of TSP from the cremators with a flue gas post-treatment system ranged from 0.5 to 70.3 mg Nm^-3^, with an average concentration of 11.0 mg Nm^-3^. The emission concentrations of PM_10_ and PM_2.5_ from the cremators without a flue gas post-treatment system ranged from 76.6 to 1,084.6 and 47.5 to 1,069.9 mg Nm^-3^, with average concentrations of 350.6 and 300.9 mg Nm^-3^, respectively. After being processed with a flue gas post-treatment system, these concentrations were efficiently reduced, ranging from 0.2 to 54.8 and 0.1 to 13.0 mg Nm^-3^, with average concentrations of 8.4 and 2.4 mg Nm^-3^, respectively. The removal rates of TSP, PM_10_ and PM_2.5_ were 97.2, 97.6 and 99.2%, respectively. These results demonstrate that dust concentrations were markedly reduced for the cremators with a flue gas post-treatment system. The reduction in PM is an effective measure to coordinate controlling the emission of PCDD/Fs [18].

CO is a product of incomplete combustion in the cremators. At furnace start-up or the initial cremation stage, a low furnace temperature can easily result in incomplete combustion. The emission concentrations of CO in the flue gas from the cremators with and without flue gas purification systems were 0.4–750.8 and 42.3–1,378.6 mg Nm^-3^, with average concentrations of 369.8 and 460.2 mg Nm^-3^, respectively. The emission concentrations of CO from the cremators with and without flue gas purification devices were 3.7 and 4.6 times the emission limits of the local standards. Because CO is predominantly regulated by the furnace temperature and the retention time of the flue gas, at the beginning of cremation, the temperature was low, incomplete combustion occurred, and the CO concentration was high. As combustion continued, the furnace temperature increased, combustion became increasingly complete, and the concentration of CO emissions decreased. Cremators with a secondary combustion chamber have a relatively low CO emission concentration. Compared to oil-fired cremators, gas-fired cremators have lower CO emissions.

SO_2_ principally originates from the combustion of sulfur in the fuel source. A CO interference experiment was performed prior to the determination of SO_2_, which was performed at the highest SO_2_ concentration and highest CO concentration. For flue gas purification systems, the deacidification device with an alkaline solution can neutralize and remove SO_2_. In this study, the emission concentrations of SO_2_ from the cremators without flue gas purification devices ranged from 3.8 to 350.2 mg Nm^-3^, with an average of 65.0 mg Nm^-3^. This level exceeded the standard limit by 116.7%, and it was also higher than the emission concentration of SO_2_ from the cremators with flue gas purification devices.

NO_x_ consists primarily of fuel-type and thermal-type gases, particularly the latter; the production of thermal-type NO_x_ increases with furnace temperature. The combustion temperature in the cremators can be up to 900~1100°C, and about 700°C in secondary chamber. In the present study, the average NO_x_ concentrations from the cremators with and without post-treatment systems were 435.5 and 280.2 mg Nm^-3^, respectively. The former value is 55% higher than the latter, which is mainly because cremators with a post-treatment system were set to a higher temperature for more complete burning and to further reduce the emission of PM and dioxins, although the side effect is more NO_X_ emissions.

Under high-temperature conditions, the strong oxidation process of combustible materials such as fuel and corpses is associated with decomposition and combination reactions of the materials. This process produces VOCs, leading to environmental pollution. The emission concentrations of VOCs from the cremators with and without flue gas post-treatment systems were in the ranges of 0.1–23.9 and 0.1–162.7 mg Nm^-3^, with average concentrations of 7.1 and 24.4 mg Nm^-3^, respectively. The former value is 70.7% less than the latter, suggesting that flue gas post-treatment systems have a particular effect on the removal of VOCs emitted by cremators.

According to the Beijing emission standard of air pollutants from crematories (DB11/1203-2015), the compliance rates of TSP, CO, SO_2_ and NO_x_ of flue gas in the monitoring samples with a post-treatment system are 87.5%, 33.3%, 87.5% and 25%, respectively, and they are 0%, 28.6%, 55.6% and 33.3% without a post-treatment system. Additionally, as shown in **Fig 2**, the compliance rates of the average emission concentrations for these four pollutants are higher for the cremators with a post-processing device than those without a post-treatment system.

### Chemical composition analysis of VOCs

**Fig 3:**
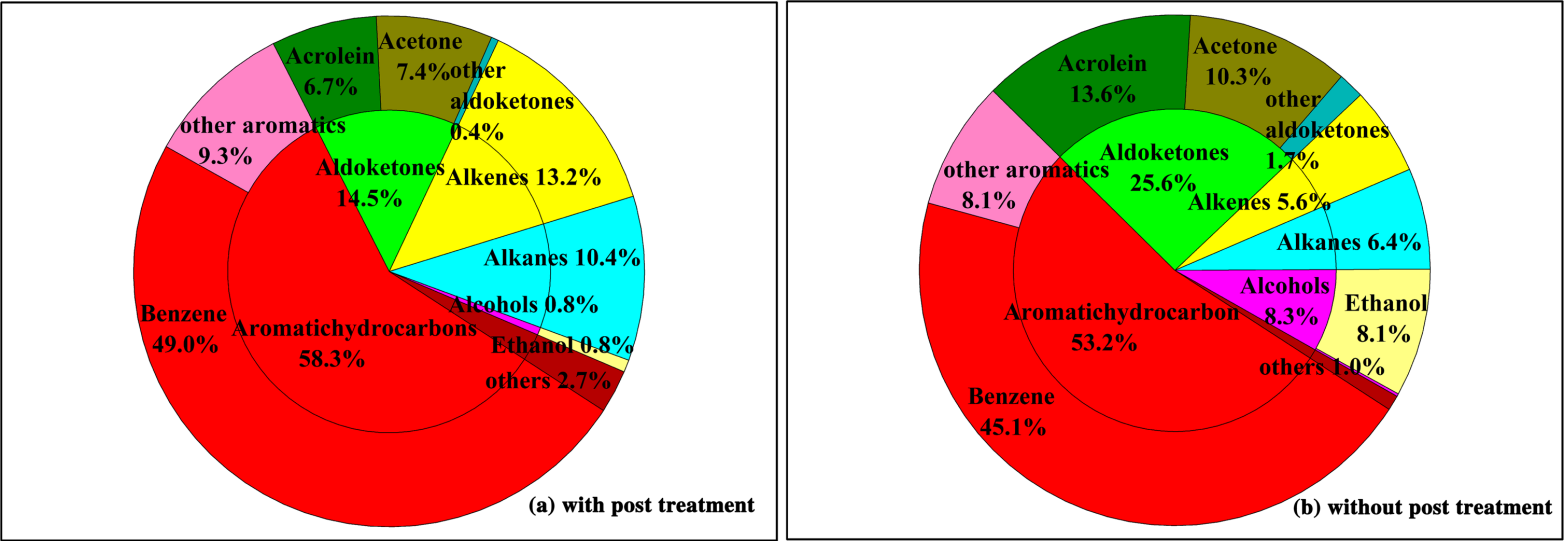
Percentage of compounds in VOCs from cremators.

The chemical components of VOC samples from the cremators were analyzed by GC-MS. Totals of 32 and 42 components were detected in the VOCs emitted from the cremators with and without flue gas purification systems, respectively. **Fig 3** displays the percentages of every chemical component in the VOCs. The top 10 components in the flue gas from the cremators without post-treatment were benzene, acrolein, acetone, ethanol, toluene, methyl chloride, propylene, 1,2-dichloroethane, 2-butanone and naphthalene, accounting for 45.1%, 13.6%, 10.3%, 8.1%, 5.5%, 3.3%, 3.2%, 1.7%, 1.6% and 1.6% of the detected VOCs samples, respectively. The top 10 components in the flue gas from the cremators with post-treatment were benzene, propylene, acetone, acrolein, toluene, 1-butene, acetonitrile, *n*-dodecane, *n*-undecane and *n*-hexane, which accounted for 49.0%, 8.1%, 7.4%, 6.7%, 5.7%, 2.9%, 2.6%, 1.6%, 1.2% and 1.1% of the detected concentrations, respectively. Based on the percentage distribution of the chemical components with and without flue gas post-treatment systems, aromatics accounted for more than 50% of the two VOC samples, and benzene, presenting high photochemical activities and severe effects on human health, accounted for approximately half of the aromatics. As shown in **Fig 3**, flue gas post-treatment systems are particularly effective at reducing the levels of aldehydes, ketones and alcohols in VOCs. The percentages of acrolein, acetone and ethanol in the VOCs of flue gas were lower for the cremators with a post-treatment system, indicating an observable removal effect of post-treatment systems for these components.

### Emission factors of flue gas from cremators

**Fig 4:**
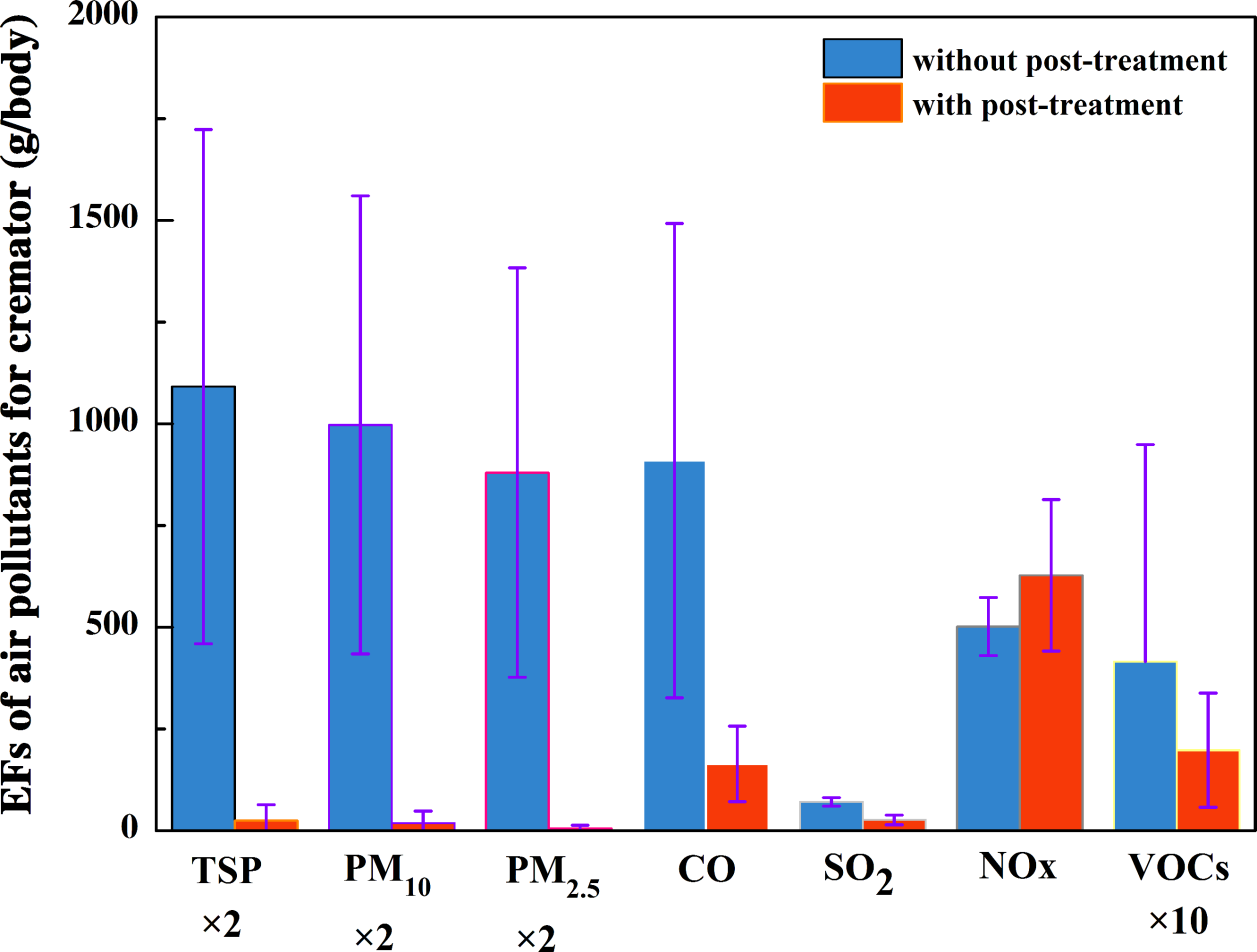
EFs of harmful air pollutants for the studied cremators.

The EFs of harmful air pollutants for the studied cremators were calculated based on practical monitoring data (data in S3 Table). **Fig 4** exhibits the characteristics of pollutant emission factors for the cremators with and without post-treatment systems based on the obtained emission factors of PM_10_, PM_2.5_ and VOCs. For the cremators with a flue gas post-treatment system, the emission factors of TSP, PM_10_, PM_2.5_, CO, SO_2_, NO_x_ and VOCs were 12.5, 9.3, 3.0, 164.1, 26.4, 627.8 and 19.8 g/body, respectively. For comparison, the emission factors of the aforementioned pollutants without a flue gas post-treatment system were 545.8, 498.7, 440.1, 909.5, 70.6, 501.6 and 41.6 g/body, respectively. Except for NO_x_, the remaining six pollutants in the post-processed flue gas were characterized by significantly lower emission factors than those in the untreated flue gas. The emission factors of TSP, PM_10_, PM_2.5_, CO, SO_2_ and VOCs were reduced by 97.7, 98.1, 99.3, 82.4, 62.6 and 52.4%, respectively. The dust removal device in the flue gas post-treatment system had a significant impact on limiting PM, and SO_2_ levels were also reduced by the deodorization spray tower with alkaline solution. The cremators with a flue gas post-treatment system were characterized by better operation management, with lower emission levels of CO than those without a flue gas post-treatment system.

According to the survey result, no activated carbon spray or adsorption occurred in the crematories. The decrease in the emission factors of VOCs may be related to the filtration-adsorption or condensation of VOCs. A combination of existing control techniques for VOC pollution, such as activated carbon adsorption and catalytic combustion measures [24], may further reduce the emissions of VOCs. However, the drawback to a flue gas post-treatment system is that the NO_x_ emission levels of cremators increased by 25.1% relative to the levels from cremators without a flue gas post-treatment system, which is likely due to the influence of temperature regulation and other factors.

**Fig 5:**
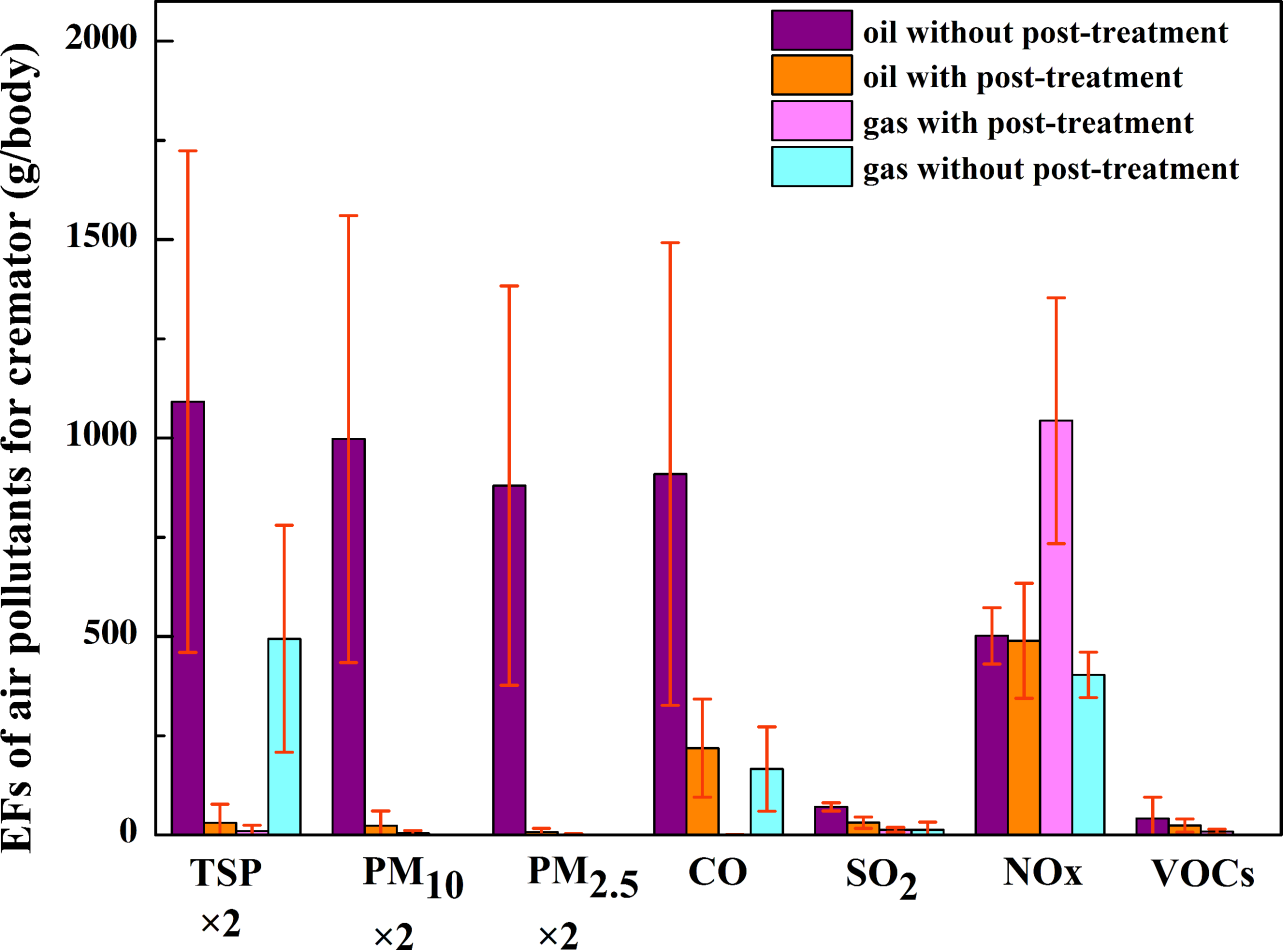
Comparison of EFs of harmful air pollutants between gas-fired and oil-fired cremators.

The emission factors for different fuel types were compared among cremators with and without post-treatment systems (**Fig 5**). During the initial discharge of air pollutants, the emission levels of various pollutants of the oil-fired cremators are higher than those of the gas-fired cremators. Regardless of the type of fuel used in the cremator, the air pollutant emission levels for those using a flue gas purification system were lower; however, due to the effect of temperature, NO_X_ emission levels were higher. The emission factors were considerably reduced for the oil-fired cremators with flue gas purification systems than those without, and the removal rates of TSP, PM_10_, PM_2.5_, CO, SO_2_ and VOCs were 97.2, 97.7, 99.2, 75.9, 56.3 and 43.4%, respectively. Similarly, the emission factors of TSP, CO and SO_2_ were considerably reduced for the gas-fired cremators equipped with a purification device. For the cremators fitted with a flue gas purification system, the emission factors were much lower for gas-fired cremators than oil-fired cremators, and the emission factors of TSP, PM_10_, PM_2.5_, CO, SO_2_ and VOCs were reduced by 68.8, 81.4, 80.1, 99.7, 58.7 and 63.4%, respectively. Therefore, a clean energy conversion of cremators from oil-fired to gas-fired could improve the combustion efficiency and reduce the incomplete combustion of corpses and fuel, effectively lowering the emission levels of pollutants such as PM, SO_2_ and CO.

### Comparison of pollutant emission characteristics with other studies

In previous investigations [9,25], the analysis results of the EFs of harmful air pollutants from cremators were 12.5–18.6 and 9.8~-4.6 mg Nm^-3^, with average concentrations of 15.6 and 17.2 mg Nm^-3^, respectively. These values are similar to the emission concentration of TSP in our study (11.0 mg Nm^-3^). However, previous studies did not report PM_10_ and PM_2.5_ levels. This is the first study in China to obtain the emission levels of PM_10_ and PM_2.5_. According to the analysis results, the percentages of PM_10_ and PM_2.5_ in the TSP of the flue gas from the studied cremators with a post-treatment system were 0.76 and 0.22, respectively, and the values for cremators without a post-treatment system were 0.89 and 0.76, respectively. This finding indicates that a PM removal device can effectively reduce the emission of inhalable particle and fine particulate matter and reduce the impact on human health and the surrounding environment.

The pollutant emission factors of the cremators obtained in this study were compared with the research results within and outside China (**Table 2**). Compared with the results mentioned in the EU EMEP/EEA guidebook (2016), the emission factors of PM, SO_2_ and NO_x_ were relatively low in the present study, which is perhaps related to the different contents of hazardous components present in the fuel source. The relatively high emission level of CO may be associated with the different control levels of combustion due to the differences among cremator device types in China and other countries as well as the operational differences in the fuel supply quantity and the fuel- and oxygen-supplying air time. Influenced by traditional customs, corpse cremation in China may also include the incineration of burial objects. Moreover, differences in flue gas treatment facilities and the standardization of their operational management may result in higher emission levels of VOCs [26].

**Table 2:** Comparison of harmful air pollutants in China and abroad (g/body).

Pollutants	Average emission factor				
	Without a post-treatment system		With a post-treatment system		
	Xue et al., 2016	This study	Xue et al., 2016	This study	EU (2016)
TSP	140.6	545.8	15.8	12.5	38.6
PM_10_		498.7		9.3	34.7
PM_2.5_		440.1		3	34.7
CO	567.8	909.5	281.4	164.1	140
SO_2_	92.7	70.6	73	26.4	113
NO_X_	189.6	501.6	134.5	627.8	825
VOCs		42		20	13

Compared with previous research results [22], the present monitoring results show that the pollutant emission levels of cremators without a flue gas post-treatment system became markedly higher, with TSP and CO increases of 288% and 60%, respectively. This is due in part to the long-term use of cremators in addition to insufficient operational maintenance, poor implementation of combustion controls and the incomplete combustion of fuel and corpses. Additionally, previous studies have primarily been based on the supervisory monitoring of cremators. To better represent the emission levels of flue gas directly discharged from the cremators, our evaluation was conducted based on the implementation of emission standards. For the cremators with a flue gas post-treatment system, the present monitoring results were much lower than those of previous data except for NO_x_. This result also reflects that adhering to stringent standardized limits and the oversight departments have strengthened the operation and maintenance of flue gas post-treatment systems and enhanced the control of combustion conditions in the cremators. By improving the combustion efficiency and reducing incomplete combustion, the pollutant emission levels of cremators can be efficiently reduced. Because of strengthened controls on combustion operating conditions, furnace temperatures and thermal-type NO_x_ generation have increased.

## Conclusions

In this study, we examined the emission characteristics of flue gas and determined the local emission factors of pollutants from cremators in Beijing, China, based on the monitoring and analysis of major air pollutants (TSP, PM_10_, PM_2.5_, SO_2_, CO, NO_x_, VOCs and their chemical components) from nine cremators.

According to the monitoring results, the pollutant emission concentrations were significantly lower for cremators with flue gas post-treatment system than those without. The pollutant emission factors of TSP, PM_10_, PM_2.5_, CO, SO_2_ and VOCs were reduced in cremators with flue gas post-treatment systems by 97.7, 98.1, 99.3, 82.4, 62.6 and 52.4, respectively. Dust removal units can effectively remove PM. Additionally, deacidification spray towers are available to remove a quantity of acidic gases and reduce SO_2_ emissions. Moreover, the operating conditions of combustion were generally optimized and adjusted in the cremators with a flue gas post-treatment system, which improved the combustion efficiency and reduced the incomplete combustion of corpses and fuel, thereby reducing pollutant emission levels.

The emissions of VOCs from cremators have been a neglected issue in previous studies, and no corresponding control requirements have been proposed for VOCs in emission standards. Based on the monitoring of the parameters in this study, we found that the process of corpse cremation produced certain emissions of VOCs, a significant source of odors emitted from crematories. Benzene is the most significant VOC, with a percentage of ~50%, and it may cause serious risks to human health. Moreover, benzene has high photochemical activity and tends to cause the secondary transformation of PM_2.5_ and ozone, which may impact air quality, meriting serious attention and concern.

Apart from the ability of flue gas post-treatment systems to reduce the emission of pollutants, clean energy conversion of the fuel types used by cremators, such as the use of natural gas in place of oil, can also effectively reduce the emissions of air fpollutants such as PM, CO and SO_2_ from cremators. Among various pollution prevention measures, control over the content of hazardous components in burial objects, the use of clean fuel and combustion optimization should be implemented in crematories to match the current requirements of stringent emission limits. Combining these measures with an efficient flue gas post-treatment system (including dust removal, deacidification and odor removal) can further reduce the emission levels of air pollutants from cremators.

## Supporting information

S1 Table Geographical coordinates for the funeral parlours sampled. (DOCX)

S2 Table Emission concentration of harmful air pollutants from cremators (mg/m^3^). (DOCX)

S3 Table Emission factors of harmful air pollutants from cremators (g/body). (DOCX)
