# Supplementary material for: Emission characteristics of harmful air pollutants from cremators in Beijing, China
Source: PLoS One. 2018 May 2;13(5):e0194226. doi: 10.1371/journal.pone.0194226 (PMC5931459; doi:10.1371/journal.pone.0194226)
Supplement: S1 Table — (DOCX) [file pone.0194226.s001.docx]

**S1 Table.** Geographical coordinates for the funeral parlours sampled

| No. | Name | X | Y |
| --- | --- | --- | --- |
| A | Babaoshan funeral parlor | 116.242844 | 39.907764 |
| B | Changping funeral parlor | 116.216789 | 40.211061 |
| C | Huairou funeral parlor | 116.650167 | 40.354003 |
| D | Pinggu funeral parlor | 117.032556 | 40.232856 |
| E | Mentougou funeral parlor | 116.109067 | 39.928442 |
| F | Daxing funeral parlor | 116.319053 | 39.572278 |
| G | Tongzhou funeral parlor | 116.661528 | 39.945531 |
| H | Dongjiao funeral parlor | 116.407442 | 39.904414 |
| I | Shunyi funeral parlor | 116.928697 | 40.195825 |
