# Supplementary material for: Emission characteristics of harmful air pollutants from cremators in Beijing, China
Source: PLoS One. 2018 May 2;13(5):e0194226. doi: 10.1371/journal.pone.0194226 (PMC5931459; doi:10.1371/journal.pone.0194226)
Supplement: S2 Table — (DOCX) [file pone.0194226.s002.docx]

**S2 Table.** Emission concentration of harmful air pollutants from cremators (mg/m^3^)

| Name | TSP | PM_10_ | PM_2.5_ | CO | SO_2_ | NO | NO_2_ | VOCs |
| --- | --- | --- | --- | --- | --- | --- | --- | --- |
| Babaoshan funeral parlor | 1.4 | 0.6 | 0.2 | 0.4 | 1.1 | 190.1 | 2.3 | 3.2 |
| Changping funeral parlor | 70.3 | 54.8 | 13.0 | 526.2 | 7.9 | 464.4 | 34.6 | 3.6 |
| Huairou funeral parlor | 3.3 | 2.8 | 1.6 | 563.4 | 5.8 | 303.3 | 0.0 | 13.7 |
| Pinggu funeral parlor | 3.5 | 2.5 | 1.3 | 39.1 | 7.8 | 117.2 | 0.0 | 16.5 |
| Mentougou funeral parlor | 154.2 | 145.9 | 136.9 | 54.4 | 6.8 | 204.1 | 0.0 | 11.3 |
| Daxing funeral parlor | 450.2 | 424.0 | 375.8 | 693.3 | 6.1 | 92.0 | 36.8 | 11.6 |
| Tongzhou funeral parlor | 116.3 | 94.7 | 74.5 | 203.8 | 2.0 | 183.3 | 18.9 | 3.7 |
| Dongjiao funeral parlor | 505.5 | 483.6 | 466.5 | 343.7 | 17.3 | 175.6 | 25.5 | 24.1 |
| Shunyi funeral parlor | 761.3 | 629.0 | 475.7 | 1378.6 | 80.4 | 164.2 | 28.9 | 84.2 |
