# Supplementary material for: Emission characteristics of harmful air pollutants from cremators in Beijing, China
Source: PLoS One. 2018 May 2;13(5):e0194226. doi: 10.1371/journal.pone.0194226 (PMC5931459; doi:10.1371/journal.pone.0194226)
Supplement: S3 Table — (DOCX) [file pone.0194226.s003.docx]

**S3 Table.** Emission factors of harmful air pollutants from cremators (g/body)

| Name | TSP | PM_10_ | PM_2.5_ | CO | SO_2_ | NO | NO_2_ | VOCs |
| --- | --- | --- | --- | --- | --- | --- | --- | --- |
| Babaoshan funeral parlor | 4.7 | 2.2 | 0.7 | 0.6 | 4.2 | 674.9 | 8.8 | 8.6 |
| Changping funeral parlor | 33.2 | 25.9 | 6.1 | 177.3 | 4.9 | 359.7 | 16.4 | 14.7 |
| Huairou funeral parlor | 3.0 | 2.5 | 1.4 | 375.1 | 5.2 | 276.7 | 0.0 | 12.3 |
| Pinggu funeral parlor | 9.2 | 6.7 | 3.5 | 103.3 | 20.7 | 310.0 | 0.0 | 43.6 |
| Mentougou funeral parlor | 402.2 | 380.6 | 357.0 | 142.0 | 17.7 | 532.4 | 0.0 | 29.4 |
| Daxing funeral parlor | 1104.2 | 1040.0 | 921.9 | 1700.5 | 45.1 | 225.7 | 90.3 | 29.2 |
| Tongzhou funeral parlor | 266.9 | 216.4 | 168.8 | 472.8 | 9.1 | 427.4 | 43.2 | 8.4 |
| Dongjiao funeral parlor | 547.6 | 506.9 | 466.7 | 250.5 | 31.3 | 183.4 | 17.9 | 20.5 |
| Shunyi funeral parlor | 407.8 | 349.5 | 286.1 | 1981.9 | 73.3 | 146.6 | 33.1 | 120.4 |
